# Tissue- and Cell Type-Specific Expression of the Long Noncoding RNA Klhl14-AS in Mouse

**DOI:** 10.1155/2017/9769171

**Published:** 2017-09-10

**Authors:** Sara Carmela Credendino, Nicole Lewin, Miriane de Oliveira, Swaraj Basu, Barbara D'Andrea, Elena Amendola, Luigi Di Guida, Antonio Nardone, Remo Sanges, Mario De Felice, Gabriella De Vita

**Affiliations:** ^1^Dipartimento di Medicina Molecolare e Biotecnologie Mediche, Università degli Studi di Napoli Federico II, Via S. Pansini 5, 80131 Napoli, Italy; ^2^Department of Internal Medicine, Botucatu School of Medicine, University of São Paulo State, Sao Paulo, SP, Brazil; ^3^Department of Medical Biochemistry and Cell Biology, Institute of Biomedicine, the Sahlgrenska Academy, University of Gothenburg, Gothenburg, Sweden; ^4^Dipartimento di Sanità Pubblica, Università degli Studi di Napoli Federico II, Via S. Pansini 5, 80131 Napoli, Italy; ^5^Biology and Evolution of Marine Organisms, Stazione Zoologica Anton Dohrn, Villa Comunale 1, 80121 Napoli, Italy

## Abstract

lncRNAs are acquiring increasing relevance as regulators in a wide spectrum of biological processes. The extreme heterogeneity in the mechanisms of action of these molecules, however, makes them very difficult to study, especially regarding their molecular function. A novel lncRNA has been recently identified as the most enriched transcript in mouse developing thyroid. Due to its genomic localization antisense to the protein-encoding Klhl14 gene, we named it Klhl14-AS. In this paper, we highlight that mouse Klhl14-AS produces at least five splicing variants, some of which have not been previously described. Klhl14-AS is expressed with a peculiar pattern, characterized by diverse relative abundance of its isoforms in different mouse tissues. We examine the whole expression level of Klhl14-AS in a panel of adult mouse tissues, showing that it is expressed in the thyroid, lung, kidney, testis, ovary, brain, and spleen, although at different levels. In situ hybridization analysis reveals that, in the context of each organ, Klhl14-AS shows a cell type-specific expression. Interestingly, databases report a similar expression profile for human Klhl14-AS. Our observations suggest that this lncRNA could play cell type-specific roles in several organs and pave the way for functional characterization of this gene in appropriate biological contexts.

## 1. Introduction

In the last few years, genome comparison across different species highlighted that, on the evolutionary scale, organismal complexity is directly correlated with the extent of the nonprotein coding (herein referred as noncoding) fraction of the total genome [1, 2]. Moreover, the discovery that the vast majority of the genome is transcribed revealed that the noncoding portion of eukaryote transcriptomes largely exceeds the protein-coding one, both in abundance and in complexity [3, 4]. These observations, together with accumulating evidences of a plethora of RNA-mediated gene regulatory networks, strongly suggested, hence, that noncoding RNAs are key players in the establishment of biological complexity [2, 5].

Noncoding RNAs are mainly classified according to their length into small and long noncoding RNAs (lncRNAs). LncRNAs are RNA molecules longer than 200 nucleotides and without any evident or conserved open reading frame. They are often 5′ capped, polyadenylated, and spliced and have been described to be expressed at levels lower than protein-coding genes but in a more tissue- and cell-specific manner [6–10]. Many of these transcripts have been reported to have potential functional orthologs in different species, despite the low sequence conservation [8, 11–13]. Although only few lncRNAs have been functionally characterized [14, 15], the number of lncRNAs identified in different cell types and correlating with different biological processes has been rising [1, 16, 17]. Such increase strongly suggests that they have multiple considerable functions, mainly mediated by their ability to associate with different classes of biomolecules. Indeed, long noncoding RNAs can be able to bind to other nucleic acids (either DNA or RNA) by base pairing to complementary sequences and/or to bind to proteins by folding in secondary structures [1, 18–20]. Nevertheless, the intrinsic pleiotropic mechanism of action of these molecules represents the major challenge in the study of the functional role of each individual lncRNA.

Recently a poorly characterized gene, reported as long noncoding RNA, emerged as the most enriched in E10.5 mouse thyroid bud transcriptome, compared to that of the whole embryo [21]. To the best of our knowledge, only few data exist in the literature about this gene, which are essentially based on genome-wide expression profiling of lncRNAs in mouse [9]. We firstly named it Thybe1 (thyroid bud enriched 1); however, the analysis of its genomic localization revealed that it is in antisense and partially overlapping with the protein-encoding gene Klhl14 in a head to head arrangement; thus, a more standard name for this gene could be Klhl14-AS. In this paper, we analyze the Thybe1/Klhl14-AS expression pattern in adult mouse tissues, both by quantitative real-time reverse-transcriptase- (qRT-) PCR and by in situ hybridization (ISH). We also identify previously unknown splicing isoforms of this gene in mouse and explore available data on human Klhl14-AS expression, thus providing an important contribution to the characterization of this novel lncRNA.

## 2. Results and Discussion

### 2.1. Genomic Organization and Identification of Alternative Transcripts of Mouse Klhl14-AS Gene

**Figure 1:**
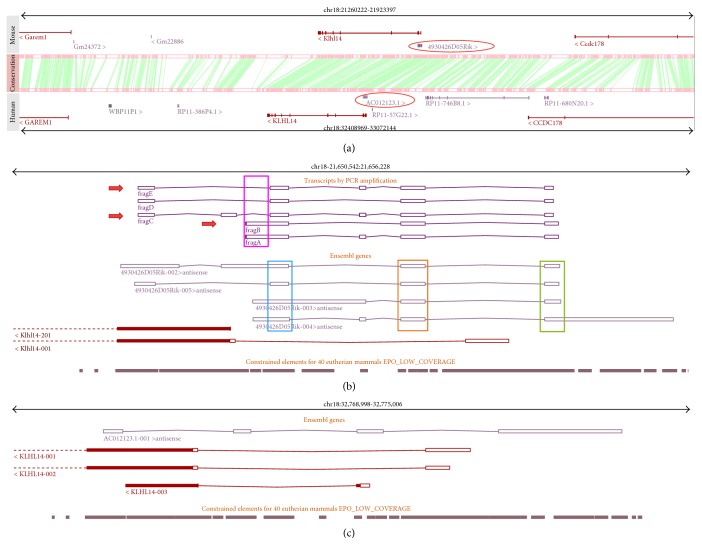
Genomic context of the Klhl14 S/AS pair in mouse and human. In (a), two genomic segments from mouse and human containing the Klhl14 S/AS pair and flanking genes are displayed. The regions are clearly syntenic, and the presence of an antisense noncoding gene (red circle) is conserved in both species. Pink boxes indicate conserved blocks, and green lines represent their correspondences. In (b) and (c), the slices are zoomed in to show the entire AS genes and the Klhl14 gene portion in overlap for the mouse (b) and human (c) genome, respectively. The constrained elements displayed as grey boxes represent regions of sequence conservation in mammals according to Ensembl. In (b), the regions shared by all the transcripts are indicated by the blue, orange, and green boxes. Fragment A to fragment E represent the isoforms identified in this study with the new ones indicated by the red arrows. The region in the pink rectangle is amplified exclusively with one set of oligos.

To shed light on the Klhl14-AS expression profile, we looked at different databases such as NCBI [22], UCSC [23], and Ensembl [24] to obtain information about gene structure and expression. In mouse genome, the gene is named 4930426D05Rik and mapped to chromosome 18qA2, as reported in Ensembl. In human genome, its ortholog is named AC012123.1 and mapped to chromosome 18q12.1. The comparison of the genomic context of mouse and human locus reveals that the regions are clearly syntenic and show high grade of conservation of blocks of sequences. In both genomes, Klhl14-AS partially overlaps with the protein-coding gene Klhl14 in a head-to-head antisense arrangement (Figure 1(a)). Mouse Klhl14-AS produces several different transcripts (Figure 1()) differing between databases both in number and in structure (see Supplementary Figure S1 available online at https://doi.org/10.1155/2017/9769171), while in human, it is reported to produce a single isoform (Figure 1(c)). We thus referred to Ensembl for further analyses, because it reports the higher number of alternative transcripts for mouse. In Figure 1(b), it is shown that mouse Klhl14-AS produces four transcripts named 4930426D05Rik 002, 003, 004, and 005. These transcripts differ for the transcription start site and/or the exons, mainly at the 5′ moiety, while three out of four show a similar 3′ end, with only Rik04 showing a longer 3′ exon. All the isoforms, however, share three regions indicated in Figure 1(b) by blue, orange, and green rectangles.

**Figure 2:**
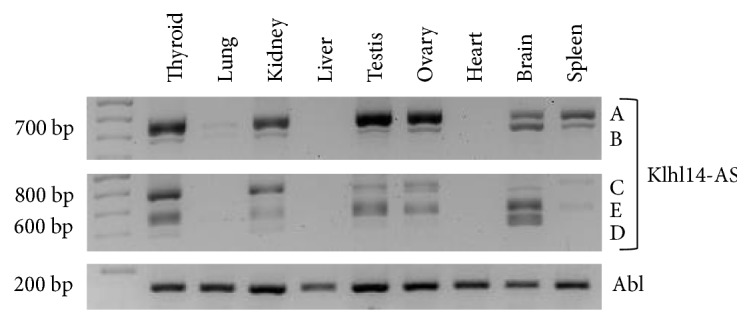
Klhl14-AS isoform expression in adult mouse tissues. Klhl14-AS transcripts were amplified by RT-PCR on total RNA from adult mouse organs. Three different PCR reactions were performed on the same template cDNA. Upper image: A and B isoforms were amplified by using the oligo set 1; middle image: C, D, and E were obtained by using oligo set 2; lower image: Abelson (Abl) amplification was performed as the control (for details see Materials and Methods). Data are representative of three independent experiments.

As Klhl14-AS was identified in the developing thyroid [21], we decided to investigate which of the reported transcripts was expressed in the adult mouse gland. We thus designed appropriate primer sets and amplified total mouse thyroid cDNA. Surprisingly, only one out of the used oligo sets, including the most internal ones, gave rise to amplicons (see Figure S2 and Table S1). This prompted us to experimentally map 5′ and 3′ ends of thyroid isoforms by performing RACE experiments. To this aim, we started from the 5′ end of the common region in the blue box and the 3′ end of the common region in the green box (Figure 1(b)), by identifying a 5′ terminus and a polyadenylated 3′ end (data not shown). Moreover, we considered also a more upstream CAGE site reported in Encode, in a region shared by most 5′ exons of the Rik002 and Rik005 isoforms. Two different primer sets were thus designed, starting from the just described putative ends. The oligo starting from the 5′ RACE sequence was used in set 1, whereas the oligo starting from the most upstream sequence reported in databases was used in set 2 (Figure S3). For each oligo set, the reverse primer was specifically designed to optimize the amplification conditions, although reverse primers are largely overlapping and both mapped very closely to the 3′ end identified by RACE. RT-PCR performed with oligo set 1 produced two products, named A and B, while that performed with oligo set 2 produced three fragments, named C, D, and E (Figure 2(), see the thyroid lane). The sequences of the isoforms amplified were mapped to the mouse genome (mm10) using BLAT (see Materials and Methods), revealing that only the two of them, A and D, correspond to the previously characterized splicing variants, Rik004 and Rik005, respectively. Importantly, we identified three novel isoforms, B, C, and E, which have not been previously annotated (Figure 1(b)). It is worth noting that we found a region that is amplified only using oligo set 1 (Figure 1(b), pink rectangle). The retention of this region only in transcripts A and B suggests that at least two different TSSs could exist for this gene. The finding that Klhl14-AS is transcribed in several different isoforms in the same organ suggests that they could play different roles through the binding of different interactors. Moreover, the presence of more than one TSS suggests that Klhl14-AS transcription could be differentially regulated in different conditions.

### 2.2. Expression Profile of Klhl14-AS in Adult Mouse Tissues

We asked if Klhl14-AS was expressed in other organs. To answer this question, we performed the above described amplifications in diverse adult mouse tissues. Such analysis indicated that Klhl14-AS transcripts are differentially expressed in different tissues and that the two used oligo sets amplify the same molecular species in all tested tissues (Figure 2). Indeed, isoforms A and B are expressed in the thyroid, kidney, testis, ovary, brain, and spleen, although at different extents. A is mostly represented in the testis and ovary, being also strongly expressed in the thyroid and kidney. B is relatively more abundant in the brain than in other organs. The transcript C is mostly expressed in the thyroid and kidney. D is weakly expressed in the thyroid and kidney, while it is highly expressed only in the brain. E is mostly represented in the thyroid, testis, ovary, and brain. It is worth noting that all the identified transcripts are expressed in the thyroid gland, whereas none of the transcripts amplified by oligo set 2 are expressed in the spleen. Lastly, the liver and heart are negative for Klhl14-AS expression, while the lung shows a weak signal only with oligo set 1. Such data reveal a tissue-specific pattern of Klhl14-AS alternative transcript expression.

**Figure 3:**
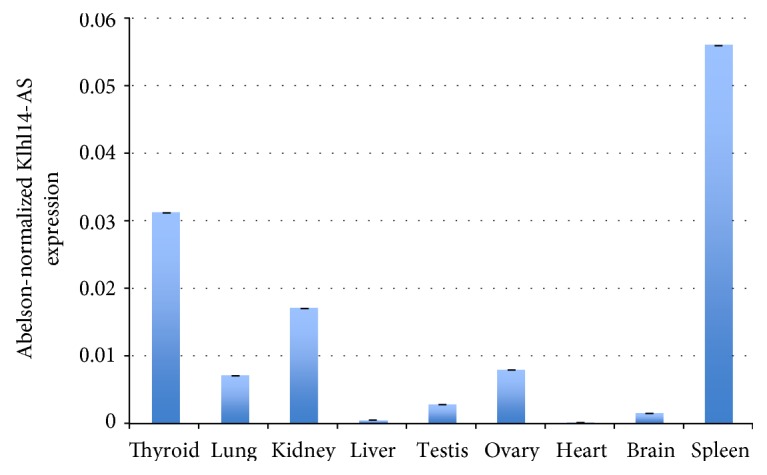
Klhl14-AS expression in adult mouse organs. Quantitative real-time RT-PCR was performed by amplifying a region shared by Klhl14-AS isoforms on total RNA from adult mouse organs. The data are reported as normalized by Abelson expression. Three replicates for each experimental point were performed. Error bars represent standard deviation of normalized ct values. Data are representative of three independent experiments.

**Figure 4:**
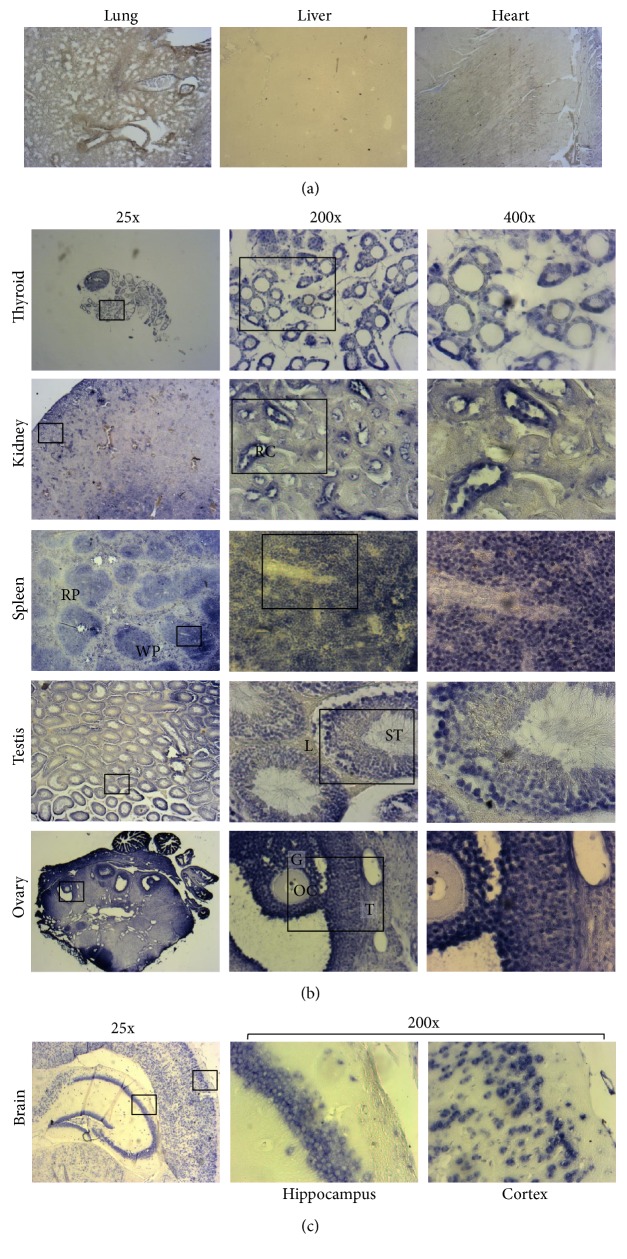
Cell type-specific expression of Klhl14-AS in adult mouse organs. In situ hybridization was performed on paraffin-embedded sections with a probe recognizing all Klhl14-AS isoforms (for details, see Materials and Methods). (a) The lung, liver, and heart are negative for Klhl14-AS staining. 25x magnifications are shown. (b) The thyroid, kidney, spleen, testis, and ovary are positive for Klhl14-AS staining. For each organ, three different magnifications are shown: 25x, 200x, and 400x. 200x magnifications correspond to the areas boxed in 25x pictures, while 400x magnifications correspond to areas boxed in 200x pictures. RC: renal corpuscle; RP: red pulp; WP: white pulp; L: Leydig cells; ST: seminiferous tubule; OC: oocyte; G: granulosa cells; T: thecal cells. All the images are reduced by 70% compared to the original ones. Data are representative of three independent experiments.

To overcome the complexity of the alternatively spliced isoform expression, we analyzed Klhl14-AS expression by using a primer set amplifying a region that is common between all known isoforms (overlapping the common splice junction between the regions included in the orange and green rectangles in Figure 1()). Whole Klhl14-AS expression among adult tissues was evaluated through qRT-PCR, showing that it is expressed in several organs at different extents. This quantitative analysis confirms that the lncRNA is not expressed in the liver and heart; weakly expressed in the brain, lung, ovary, and testis; and more strongly expressed in the kidney, spleen, and thyroid gland (Figure 3()). However, qRT-PCR, such as the standard PCR previously performed, measures the RNA in the whole organ, thus missing the possible differences between different cells types in the same organ. This technical limit could lead to underestimating Klhl14-AS presence in specific cell types. To deal with this problem, we performed ISH analysis by using a Klhl14-AS-specific probe that allowed to clearly define the lncRNA distribution in specific cellular types. A sense probe was used as a control (Figure S4). ISH performed on adult mouse organs, while confirming qRT-PCR results, revealed that Klhl14-AS is expressed in a cell type-restricted manner in the context of each organ. As expected, Klhl14-AS staining is absent in the liver and heart (Figure 4(a)). Unexpectedly, Klhl14-AS staining is absent also in the lungs (Figure 4(a)), where the qRT-PCR showed a detectable although weak positivity (Figure 3()). One possible explanation is that ISH has a lower detection capability than qRT-PCR; thus, a weak level of expression could be undetectable by ISH. Conversely, all other organs positive for Klhl14-AS expression by qRT-PCR show a clear signal by ISH, with differences in the distribution between specific cell types (Figure 4(b)). In the thyroid, the lncRNA is highly expressed in follicular cells, representing the epithelial component of thyroid follicles. In the kidney, Klhl14-AS is mainly expressed in the renal corpuscles of the cortex (see 200x magnification). In the spleen, it is expressed in the rounded areas of white pulp consisting of lymphoid cells, while it is not detected in the red pulp (see 25x magnification). In the testis, the lncRNA is expressed in the seminiferous tubules, while resulting negative in the Leydig cells of the intertubular connective tissue (see 200x magnification). In the ovary, Klhl14-AS is expressed in the follicles, showing a strong signal in granulosa cells of the preovulatory follicles, while it was negative in the oocyte and theca cells (see 200x magnification).  Figure 4(c) shows the ISH of the brain, where Klhl14-AS expression is strongly detected in the hippocampus and the in cortex (see 200x magnification).

We also did a survey of existing data about the human Klhl14-AS ortholog expression pattern. Gtex annotations in the UCSC database, obtained by RNA sequencing, report that the lncRNA is strongly expressed in the thyroid but weakly expressed in the spleen, testis, kidney cortex, and fallopian tube (Supplementary Figure S5) (http://bit.ly/klhl14-as). The expression pattern of human Klhl14-AS overlaps in part to that shown here in mouse, at least for the organs with the highest expression levels. The differences observed for some organs (i.e. brain, ovary) could be due either to the different experimental approaches or to real differences between the two species.

The conservation of Klhl14-AS sequence and genomic organization, together with a partially overlapping expression pattern, is suggestive of an important biological role of Klhl14-AS.

## 3. Conclusion

Identifying the biological role and the mechanism of action of long noncoding RNAs is among the most challenging issues in functional genetics studies, where the setting of appropriate experimental models is a critical issue, given their tissue specificity. Klhl14-AS was the first lncRNA identified in the thyroid gland, and here, we demonstrate that it is also expressed in very different tissues such as those of the kidney and brain. Moreover, we identify previously uncharacterized Klhl14-AS alternatively spliced transcripts and describe the differential expression pattern in several organs, suggesting that each isoform could play a specific role in a given physiological context. Indeed, the functional specificity of Klhl14-AS could arise by the combination of different variants with cell type-specific interactors that likely take place in different tissues. Taken together, our data represent the starting point for the characterization of this lncRNA, which is likely to be involved in relevant biological phenomena and possibly in different diseases.

## 4. Materials and Methods

### 4.1. Animal Experiments

All animal experiments were performed in accordance with the Italian and European guidelines and were approved by the local Ethical Committee and by the Italian Ministry of Health. Animals were maintained under specific pathogen-free conditions in the animal house facility of the Dipartimento di Medicina Molecolare e Biotecnologie Mediche. A total of eight wild-type C57BL/6 mice of both sexes were used for both molecular and histological analyses.

### 4.2. Transcript Mapping

5′ and 3′ ends were investigated with the use of 3′ RACE System (Invitrogen 18373-027) and 5′ RACE System (Invitrogen 18374-041) according to the manufacturer's specifications.

cDNA from adult mouse tissues were amplified with the Pwo SuperYield DNA Polymerase (Roche 04 340 850 001) using oligos representing the ends obtained through RACE experiments and the 5′ oligo designed on the second upstream CAGE site reported in UCSC—Klhl14-AS CAGE II: CGCGTACTGCATGCGGGTCTCA, Klhl14-AS 5′ RACE: GAGAGAGGAACAACAATCAAGGC, Klhl14-AS 3′ RACE: GGGGATTAGAGTTTATTTTTGTCATCTC, and Klhl14-AS 3′ RACE inner: ATTCATCCAGATCACAGCTAAG.

The sequences from the identified isoforms in FASTA format were mapped to the mouse genome (mm10) using BLAT (https://genome.ucsc.edu/cgi-bin/hgBlat), and the results were exported in PSL format. The results in PSL format were uploaded as a custom track in the UCSC browser and converted into GTF using the UCSC table browser. The genome browser images were generated using the Ensembl genome browser (v88) for human (GRCh38.p10) and mouse (GRCm38.p5) where a custom track was created and uploaded to visualize the PCR fragment mappings in GTF format on the mouse genome. The genome browser screenshots were exported in PDF format, and processing of the vector graphics was done in Inkscape (v0.48.4).

### 4.3. Quantitative Real-Time PCR

Total RNA was isolated from mouse organs as previously described [25] using TRIzol® Reagent (Invitrogen 15596026) according to the manufacturer's specifications. Each sample corresponds to a single organ except the thyroid. Due to the small size of mouse thyroid, two glands were pooled for total RNA extraction. Total cDNA was generated with the SuperScript® III First-Strand Synthesis System for RT-PCR (Invitrogen 18080051), according to the manufacturer's specifications. Real-time PCR on total cDNA was performed with iTaq™ Universal SyBR® Green Supermix (Bio-Rad 172-5124) using gene-specific oligos—Klhl14-AS F: GGCTCCTCTCCACTCACTTTC, Klhl14-AS R: TCAGCTCAGCAGCGAAGTC, Abelson F: TCGGACGTGTGGGCATT, and Abelson R: CGCATGAGCTCGTAGACCTTC.

### 4.4. In Situ Hybridization

Organs were fixed in 4% PFA (overnight, 4°C), washed in saline solution, dehydrated in solutions at increasing ethanol concentration from 70% to 100% (overnight, 4°C), and paraffin-embedded at 60°C after xylene soaking. The period of each step is determined according to the size of the processed sample.

Paraffin-embedded samples were sliced in 7 *μ*m sections and analyzed. To perform the in situ hybridization, the sections were deparaffinized in xylene and rehydrated with EtOH 100% to EtOH 50%. After rehydration, the hybridization was performed as described in Fagman et al. [21], using a specific probe for Klhl14-AS amplified with Pwo SuperYield DNA Polymerase from adult mouse thyroid cDNA using the following oligos—Klhl14-AS sp6: GGCTGAACAGGAAGGGACCCT and Klhl14-AS T7: CAGATCACAGCTAAGAAAAAAGC.

PCR product was purified using the USB® PrepEase® Gel Extraction Kit (Affymetrix 78756). Digoxigenin-labelled riboprobes (sense and antisense) were obtained using the DIG-labeling RNA kit (Roche Diagnostics Basel, Switzerland) following the manufacturer's instructions. No signal was detected with the sense riboprobes (not shown). Images were obtained using an Axioskop microscope equipped with an Axiocam 105 color digital camera (Zeiss, Oberkochen, Germany). Images were processed using the Axion Vision software.

## Supplementary Material

The information of supplementary materials are as follows: Figure S1. (A) NCBI representation of mouse (upper panel) and human Klhl14-AS locus (lower panel) . Mouse Klhl14-AS shows two different transcripts, whereas a single transcript is reported for the human gene. The releases for the mouse and human genome are 106, 2016-06-22 and 108,2016-06-07 respectively. (B) UCSC data base reports one transcript both for mouse (upper panel) and human (lower panel) Klhl14-AS. The data refer to the versions GRCm38 /m10 and GRCh38 /hg38 for mouse and human genomes respectively. Figure S2. Amplification of Klhl14-AS predicted isoforms. (1) Four different primers were used. One forward primer was designed on the most upstream reported CAGE site (red arrow), while the other was designed on the alternative reported transcription start site (green arrow). The reverse primers were designed on the two predicted 3′ ends (blue and pink arrows) of the reported transcripts. (2) Scheme of the different combinations of oligos used to amplify Klhl14-AS isoforms. (3) PCR results: three different bands were obtained with the oligo set B, while the amplifications with sets A and C did not give any products. Primers sequence are reported in table S1. Table S1. Sequences of the primers reported in Figure S2. Most upstream 5′ (CAGE I, red arrow in figure S2), inner 5′ end (INNER 5′, green arrow in figure S2), upstream 3′ (UPSTREAM 3′, blue arrow in figure S2), most downstream 3′ reported end (DOWNSTREAM 3′, pink arrow in figure S2). Figure S3. Two different oligo sets were used to map thyroid Klhl14-AS transcripts. The two oligo sets differ mainly for the forward oligo used: for oligo set 1 (red arrows) the forward primer was designed starting from one of the CAGE sites reported in UCSC, while for oligo set 2 (blue arrows) the forward primer was designed starting from the 5′ end identified by RACE. Reverse primers instead were both designed starting from the most 3′ sequence identified by RACE, by slightly shifting the sequence to optimize melting temperature and amplification efficiency. Figure S4. Klhl14-AS sense probe were used as negative control. In situ hybridization performed on paraffin-embedded sections with a sense probe for Klhl14-AS. 200X magnification of thyroid and 25X magnification of brain sections are shown. Both images are reduced by 25% compared to the original ones. Figure S5. Human Klhl14-AS expression pattern partially overlaps with the mouse one. UCSC annotated Bioinformatic analysis from Gtex RNA-seq data shows that human Klhl14-AS is expressed in fallopian tube, kidney cortex, spleen, testis and thyroid (bit.ly /klhl14-as).
