# Supplementary material for: Tissue- and Cell Type-Specific Expression of the Long Noncoding RNA Klhl14-AS in Mouse
Source: Int J Genomics. 2017 Sep 10;2017:9769171. doi: 10.1155/2017/9769171 (PMC5610845; doi:10.1155/2017/9769171)
Supplement: Supplementary file 1 — The information of supplementary materials are as follows: Figure S1. (A) NCBI representation of mouse (upper panel) and human Klhl14-AS locus (lower panel) . Mouse Klhl14-AS shows two different transcripts, whereas a single transcript is reported for the human gene. The releases for the mouse and human genome are 106, 2016-06-22 and 108,2016-06-07 respectively. (B) UCSC data base reports one transcript both for mouse (upper panel) and human (lower panel) Klhl14-AS. The data refer to the versions GRCm38 /m10 and GRCh38 /hg38 for mouse and human genomes respectively. Figure S2. Amplification of Klhl14-AS predicted isoforms. (1) Four different primers were used. One forward primer was designed on the most upstream reported CAGE site (red arrow), while the other was designed on the alternative reported transcription start site (green arrow). The reverse primers were designed on the two predicted 3′ ends (blue and pink arrows) of the reported transcripts. (2) Scheme of the different combinations of oligos used to amplify Klhl14-AS isoforms. (3) PCR results: three different bands were obtained with the oligo set B, while the amplifications with sets A and C did not give any products. Primers sequence are reported in table S1. Table S1. Sequences of the primers reported in Figure S2. Most upstream 5′ (CAGE I, red arrow in figure S2), inner 5′ end (INNER 5′, green arrow in figure S2), upstream 3′ (UPSTREAM 3′, blue arrow in figure S2), most downstream 3′ reported end (DOWNSTREAM 3′, pink arrow in figure S2). Figure S3. Two different oligo sets were used to map thyroid Klhl14-AS transcripts. The two oligo sets differ mainly for the forward oligo used: for oligo set 1 (red arrows) the forward primer was designed starting from one of the CAGE sites reported in UCSC, while for oligo set 2 (blue arrows) the forward primer was designed starting from the 5′ end identified by RACE. Reverse primers instead were both designed starting from the most 3′ sequence identified by RACE, by [file 9769171.f1.pdf]

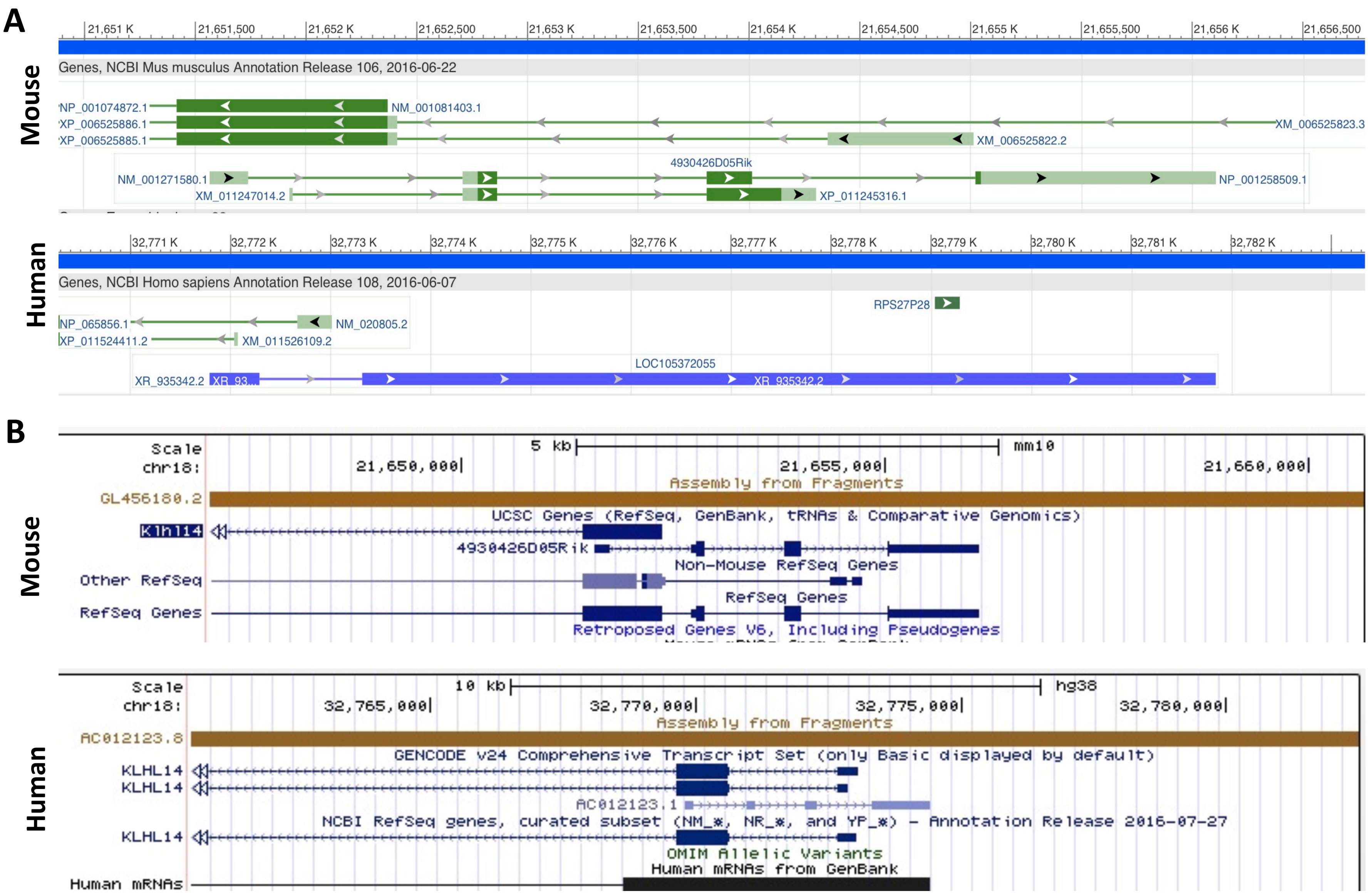

Figure S1. **(A)** NCBI representation of mouse (upper panel) and human Klhl14-AS locus (lower panel) . Mouse Klhl14-AS shows two different transcripts, whereas a single transcript is reported for the human gene. The releases for the mouse and human genome are 106, 2016-06-22 and 108,2016-06-07 respectively. **(B)** UCSC data base reports one transcript both for mouse (upper panel) and human (lower panel) Klhl14-AS. The data refer to the versions GRCm38/m10 and GRCh38/hg38 for mouse and human genomes respectively

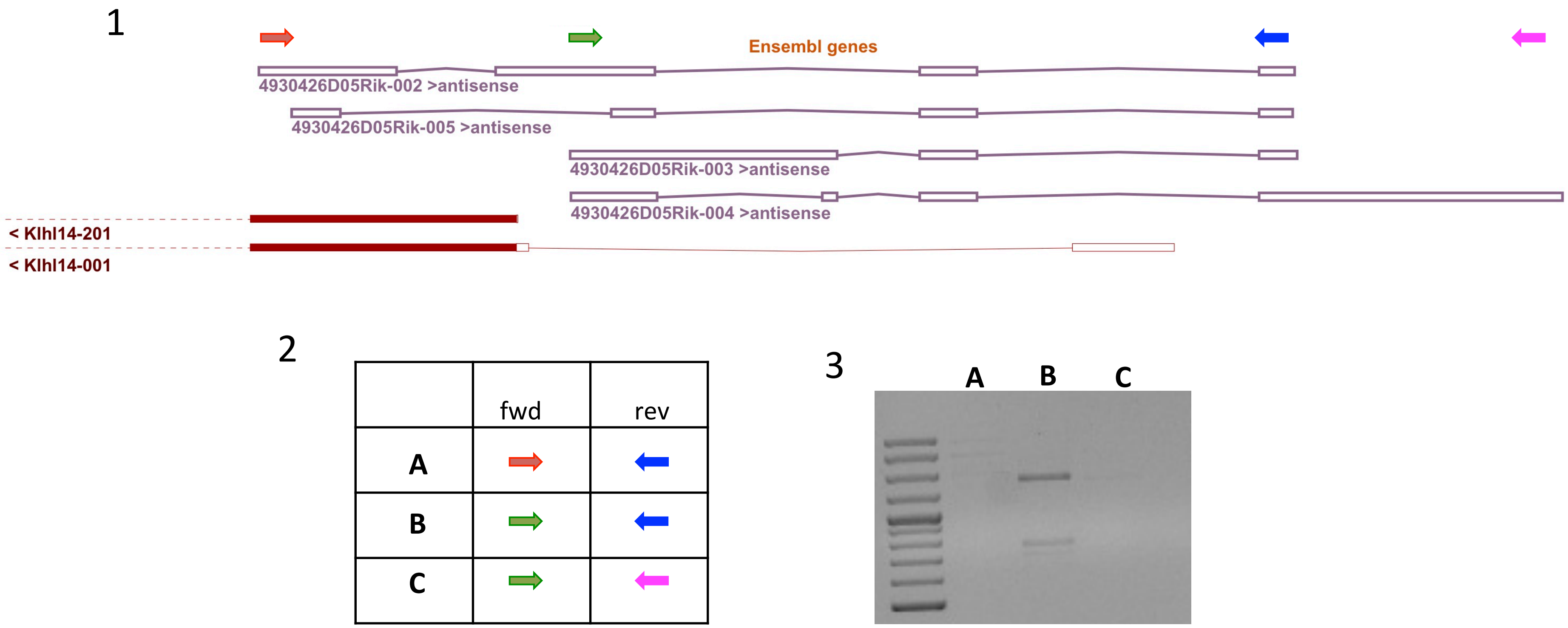

Figure S2. **Amplification of Klhl14-AS predicted isoforms.** (1) Four different primers were used. One forward primer was designed on the most upstream reported CAGE site (red arrow), while the other was designed on the alternative reported transcription start site (green arrow). The reverse primers were designed on the two predicted 3’ ends (blue and pink arrows) of the reported transcripts. (2) Scheme of the different combinations of oligos used to amplify Klhl14-AS isoforms. (3) PCR results: three different bands were obtained with the oligo set B, while the amplifications with sets A and C did not give any products. Primers sequence are reported in table S1.

Primer name	Primer sequence
CAGE I	GTAGTTCATGGCGTCCAGCAG
INNER 5’	AAGAGGGAGGTGTGAAGG
UPSTREAM 3’	GGGGATTAGAGTTTATTTTTGTCATCTC
DOWNSTREAM 3’	AAGAGGGAGGTGTGAAGG

Table S1. **Sequences of the primers reported in Figure S2**. Most upstream 5’ (CAGE I, red arrow in figure S2), inner 5’ end (INNER 5’, green arrow in figure S2), upstream 3’ (UPSTREAM 3’, blue arrow in figure S2), most downstream 3’ reported end (DOWNSTREAM 3’, pink arrow in figure S2).

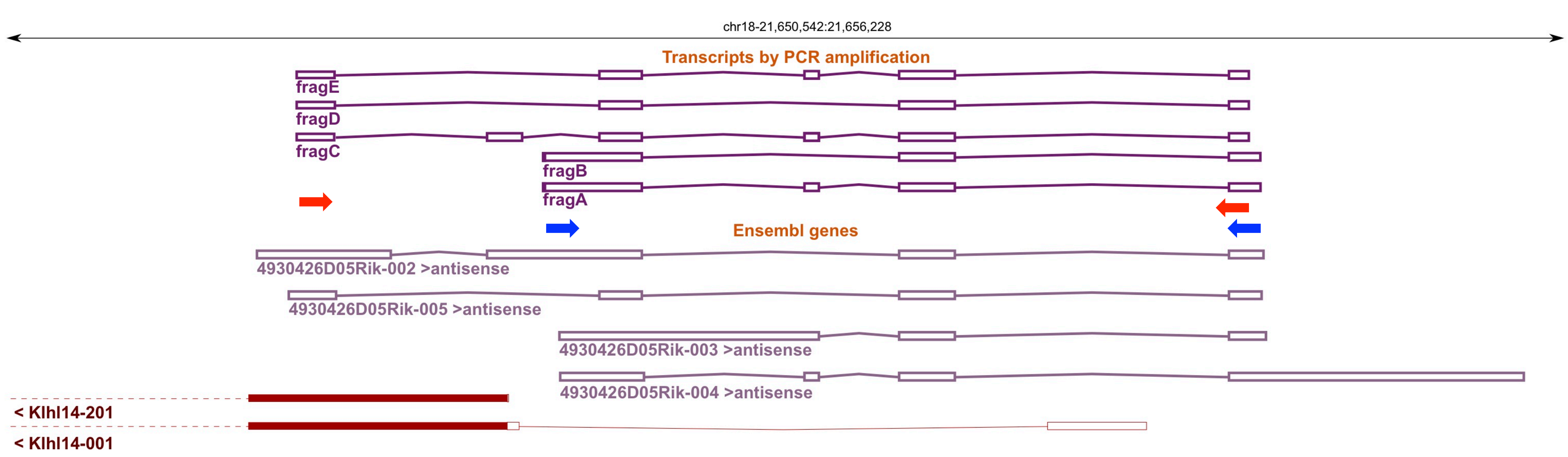

Figure S3. **Two different oligo sets used to map thyroid Klhl14-AS transcripts.** The two oligo sets differ mainly for the forward oligo used: for oligo set 1 (red arrows) the forward primer was designed starting from one of the CAGE sites reported in UCSC, while for oligo set 2 (blue arrows) the forward primer was designed starting from the 5' end identified by RACE. Reverse primers instead were both designed starting from the most 3' sequence identified by RACE, by slightly shifting the sequence to optimize melting temperature and amplification efficiency.

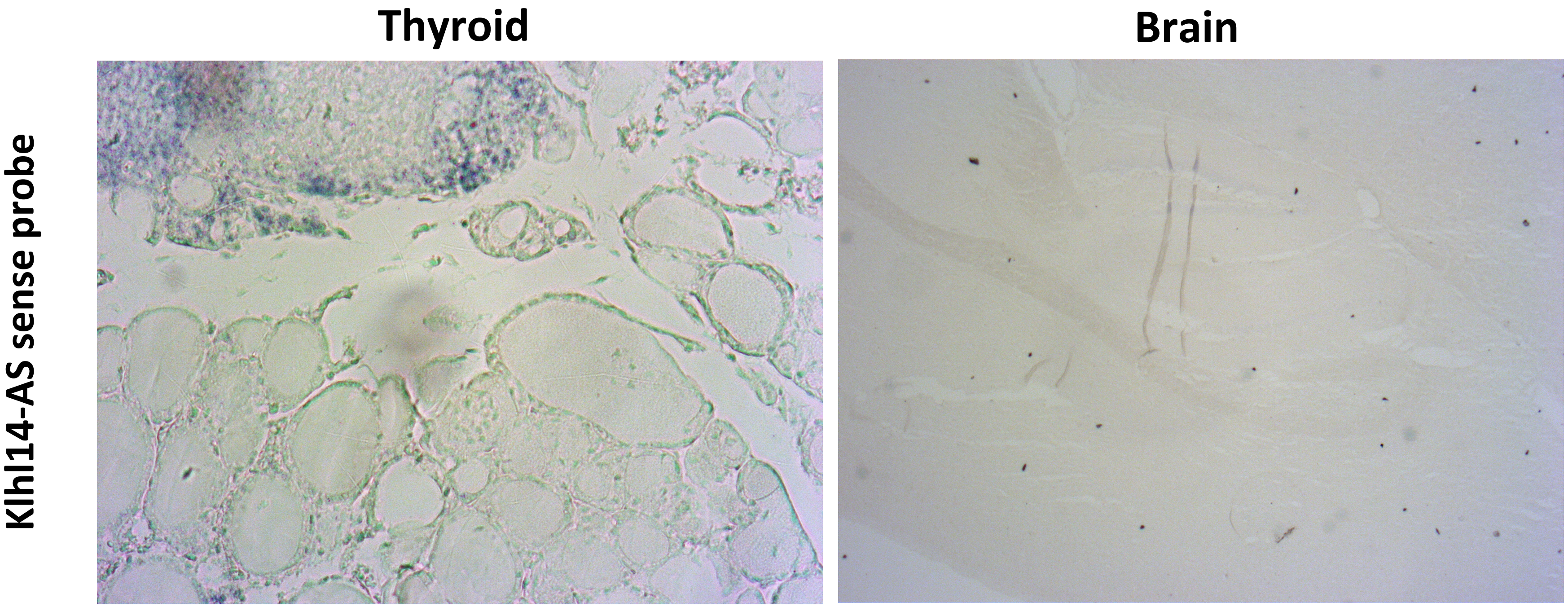

Figure S4. Klhl14-AS sense probe used as negative control. In situ hybridization performed on paraffin-embedded sections with a sense probe for Klhl14-AS. 200X magnification of thyroid and 25X magnification of brain sections are shown. Both images are reduced by 25% compared to the original ones.

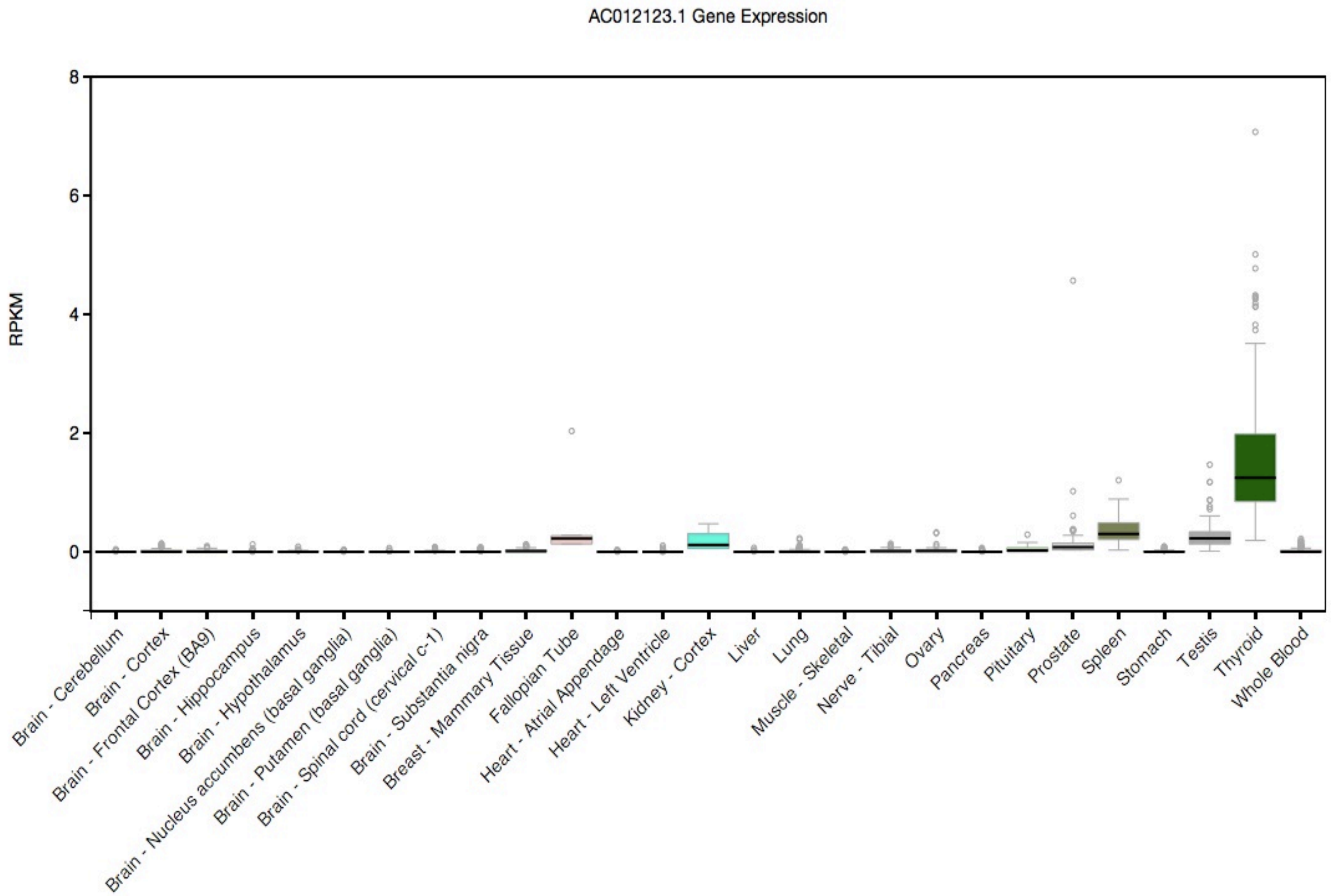

Figure S5. Human Klhl14-AS expression pattern partially overlaps with the mouse one. UCSC annotated Bioinformatic analysis from Gtex RNA-seq data shows that human Klhl14-AS is expressed in fallopian tube, kidney cortex, spleen, testis and thyroid (bit.ly/klhl14-as).
